# Image Segmentation Based on Dynamic Particle Swarm Optimization for Crystal Growth

**DOI:** 10.3390/s18113878

**Published:** 2018-11-11

**Authors:** Yu Li, Shouyu Wang, Jialin Xiao

**Affiliations:** Department of Electronic and Communication Engineering, East China University of Science and Technology, Shanghai, China, 200237; liyu@ecust.edu.cn (Y.L.); y30180629@mail.ecust.edu.cn (J.X.)

**Keywords:** crystal growth, image segmentation, dynamic particle swarm optimization (DPSO), threshold band

## Abstract

In order to realize the intelligent production of sapphire crystal, it is important to obtain the growth status from the furnace by charge coupled device (CCD). However, a significant challenge is that traditional approaches are often not valid to separate the images of the melting interface well due to the low contrast and uneven brightness from the heater. In this paper, an improved Otsu algorithm based on dynamic particle swarm optimization (DPSO) is proposed to find the exact threshold band as contour of the crystal. In this method, the Otsu method is constructed firstly, then DPSO is used to find the optimal threshold band. Experimental results show that the proposed algorithm can separate the texture of crystal growth images well and has high robustness.

## 1. Introduction

Sapphire crystal with excellent thermal properties, optical properties, and mechanical properties has important scientific research value and wide market prospect [1,2,3], such as excellent light-emitting diodes of the substrate material etc. However, the quantity of natural sapphire is rare and its purity is low. Hence, it cannot meet the great demand of the industry and other fields. Therefore, it is important to efficiently produce mass sapphire by artificial synthesis.

The growth of artificial crystal is usually finished in the melting furnace through the process of melting, seeding, crystallizing, and cooling [4], and the whole process depends on the temperature distribution of the melt surface. Because the melting point of sapphire crystal is up to 2045 °C, tungsten rods are used to heat the crucible furnace, and the maximum electric current through the tungsten rods is more than 7000 A when the equipment is working. Hence, it is difficult to measure the temperature in the furnace by sensors directly or set up pyrometers for obtaining the temperature distribution. Therefore, it is indispensable to develop technologies to obtain the status of crystal growth and it is a good method to measure the temperature distribution by machine vision [5]. Thus, the operators could judge the growth status of the crystal in the stove by human-machine interface rather than observing the melt surface by their naked eyes, since long observation of the melt surface is harmful to the eyes. A CCD image sensor, produced by Hitachi Corp. in Japan, is used here for image acquisition. The images obtained by this sensor are of good quality. In fact, images can be affected by a number of factors. Due to the positions of the tungsten rods, heat waves from the tungsten rods arranged on the edge are not coherent in every direction and the light is strong in the furnace. Under this environment, the image of objective and background is very close and it causes the contrast degree of the images obtained by CCD to be low. This special signal environment and high temperature of more than 2000 °C will lead to low contrast and resolution of CCD images. In addition, uneven and random interference, such as oil mist and noise from the heater, will often make the texture of the melting interface obscure. Obviously, these all make it difficult to form a correct description of the status of crystal growth.

**Figure 1:**
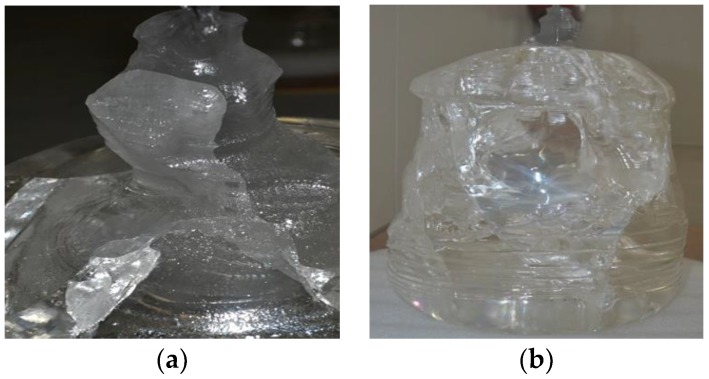
Images of crystal defects. (**a**) Polycrystallinity; (**b**) Cracking and air bubbles.

The temperature distribution of the melting interface in the seeding step has an important influence on product quality [6]. The purpose of seeding is to remove impurities and dislocation [7]. For this reason, the key is to find the right growth temperature for the sapphire and to develop fine seeds. As shown in Figure 1, improper operation will directly lead to crystal defects, including polycrystallinity, cracking, air bubbles, and so on. Therefore, it is necessary to collect information of the melting interface by CCD and to judge the growth status by the segmented images in real-time [7]. Obviously, it is also valuable to realize automatic and large-scale production.

To obtain the growth status of sapphire correctly and quickly, image segmentation is no doubt an important topic, and thus it is the emphasis of this paper. Many techniques have been developed for image segmentation. Among them, the principle of similarity has been widely used, which utilizes the similarity among the image objects with pre-defined criteria for partitioning. Thresholding, edge detecting, region splitting, and clustering are similarity-based methods [8,9]. In recent years, increasing importance has been given to novel technologies such as the fractal-wavelet technique in image segmentation [10,11].

### 1.1. Methods Based on Threshold Segmentation 

Threshold segmentation of digital images is one of the widely used techniques for image segmentation. It divides a digital image with several or fixed threshold values. In the same kinds of pixels, objects with same gray value are regarded as the same object [8]. The characteristics of this method are simple and convenient; it does not need prior information of the image. However, lighting and noise will affect the determination of the threshold. Image information may be lost if the gray value of the target is not far from that of the background. Hence, it does not work well for an image without any obvious peaks or with broad and flat valleys [12].

### 1.2. Methods Based on Edge Detection Segmentation 

Edge detection can realize image segmentation by recognizing and extracting the features of the image boundary and can detect image contours. The image obtained after edge segmentation well retain the image’s morphological features. In the region of the image edge, the gray value changes significantly. On the contrary, in the edge of the area, the image gray value changes little [9]. Thus, a derivative of the maximum or the zero point of the second derivative can be used to determine the boundary of the image. The first order differential operators include the Roberts operator, Sobel operator, Prewiit operator, etc. The second order differential operators include the Laplacian operator, Kirsh operator, and so on. The advantage of edge detection algorithms is the convolution operation between template and image, which greatly reduces the time complexity. It works well for images with good contrast between regions and it does not work well with images in which the edges are ill-defined or too numerous [12]. In order to minimize the impact of noise on image segmentation, image smoothing is often required in these methods.

### 1.3. Methods Based on Region Segmentation 

Region segmentation divides an image into different regions according to the similarity criteria. Methods based on common regional segmentation include the regional growth method, regional division combination method, and watershed method. The key idea of this method is to select the right seed points and determine the right growth rules [8]. The advantage of regional segmentation is that it considers not only the similarity of pixel grayscale values, but also the spatial adjacency of pixel points. It works best when the region homogeneity criterion is easy to define. However, region growing has inherent dependence on the selection of the seed region and the order in which pixels and regions are examined. The segments resulting from region splitting appear too square due to the splitting scheme [12].

### 1.4. Methods Based on Clustering Segmentation 

The clustering method divides the sample data into different categories according to the similarity of measurement criteria However, there are two problems in this method. One is how to compare the similarity between sample data, and the other is how to divide these data into different categories according to their similarity [13]. The main advantage of this method is its strong adaptability to various images, while the disadvantages are that it can fall into local optimization and has poor noise resistance.

Considering the above existing segmentation methods, thresholding holds the prime position from the view of robustness, simplicity, and accuracy [14]. Thresholding-based segmentation subdivides an image into smaller segments, using at least one gray level value to define its boundary. However, it is difficult to choose the threshold values because gray level histograms of real-world images are more complex than bi-model grey level histograms.

Over the past few years, many thresholding-based segmentation techniques have been reported [15,16,17]. Among them, Otsu’s method, based on the principle of between-class variance, has been proven to be one of the best thresholding methods [15]. To decide the optimal threshold values, maximizing the between-class variance of the histogram is used in this method. The conventional Otsu method is often proposed to solve bi-level thresholding problems. However, this method suffers a serious drawback of exponential growth in computing complexity. Hence, it cannot be practically extended to multilevel thresholding problems. Several methods have been reported to improve efficiency and reduce complexity. Typically, recursive algorithms can reduce the long processing time to determine the optimum threshold values with the help of a lookup table [16,17]. But, at the same time, these methods still suffer from increasing computational time as the number of thresholds increases.

In order to overcome the above problems, some methods based on swarm intelligence have been proposed in recent years. These methods refer to utilizing the behavior characteristics of individuals with simple intelligence through cooperation and organization. They have naturally distributed and self-organization characteristics. Research on swarm intelligence has existed for many years, and several important results have been achieved. Evolutionary algorithms such as genetic algorithms (GA) [18,19], ant colony optimization [20], particle swarm optimization (PSO) [21,22], and bacterial foraging algorithm (BF) [23] are commonly used in image segmentation based on multilevel thresholding. Among these algorithms, GA, PSO, and BF are more popular. PSO algorithms are inspired by the intelligent behaviors of bird flocking to find the optimal thresholds [24]. However, PSO shows some problems, such as the inability to find global optimization values, low convergence speed, and so on. Bacterial foraging is motivated by the foraging behavior of *Escherichia coli* presented in the human intestine. The BF algorithm provides better performance based on the quality of the solution and the speed of convergence than the other existing multilevel thresholding methods. However, the efficiency and the robustness of this algorithm are related to the chemotaxis step size. Large step size accelerates the progress of searching for the optimum position but does not ensure the global optimum. On the other hand, small chemotaxis step size guarantees that bacteria will find the global optimum, but it requires more searching time. Meanwhile, a few state-of-the-art methods have been developed for multilevel thresholding based on bio-inspired computing paradigm, such as the Grey Wolf optimizer [25], Krill Herd Optimization [26], Whale Optimization, and the modified firefly algorithm [27]. In [28], a novel thresholding extraction method based on variational mode decomposition (VMD) is used to non-recursively decompose a histogram into several sub-modes for minimizing Otsu’s objective function. Recently, a multilevel thresholding technology using adaptive wind driven optimization was proposed [29]. Though the above methods perform satisfactorily, achieving the best values for the objective function still requires high computational cost.

The image segmentation of a melting interface is essential to decide the status or correct seeding time for intelligent crystal growth equipment. However, strong heat radiation and turbulent flow fields in the high-temperature and high-luminance environment inevitably interfere with the CCD camera signal. This causes the target image to become blurred and the images of the melt interface to have poor texture. Hence, it is difficult to obtain good performance by traditional methods. Inspired by the fact that the grey value of the boundary of different regions is different due to the uneven illumination, and the grey value of the crystal image is continuously distributed, an improved Otsu algorithm based on dynamic particle swarm (DPSO) is presented for image segmentation during crystal growth. Firstly, particles are distributed in different areas, and different areas will undergo local optimization. Secondly, parallel processing of the proposed algorithm reduces the computing time and improves the performance. Then, image enhancement is adopted to enlarge the grey difference between crystal and boundary. This method maximizes the Otsu algorithm’s objective function using DPSO to find the optimal threshold band. After getting the threshold band, the given growth images are subdivided into small segments based on the thresholds.

The remainder of the paper is organized as follows: in Section 2, a brief review of the Otsu algorithm and the standard PSO is presented. Then, Section 3 describes the proposed method in detail. Then, experimental analysis and results are discussed in Section 4. Finally, conclusions are drawn in Section 5.

## 2. Theoretical Background

### 2.1. Image Segmentation Based on the Otsu Algorithm

Otsu is a classical algorithm of image segmentation; an image can be divided into object region (A) and background region (B). To get the optimal threshold, the difference between the object and background needs to be as great as possible. The measurement of that difference is called Otsu [15].

Let *L* stand for the number of grey levels of a given image f(x,y), so the intensity values are in the range (0, L−1). Define $P_{j}=\frac{n\left(j\right)}{M},$ where n(j) denotes the number of pixels with grey value and j and M represent the total number of image pixels. Then, Otsu’s method can be defined as

(1) $$Max\left[f\left(t\right)=j_{0}+j_{1}\right]$$

where,

$$\varphi_{0}=\omega_{0}{\left(\mu_{0}-\mu_{T}\right)}^{2},\;\varphi_{1}=\omega_{1}{\left(\mu_{1}-\mu_{T}\right)}^{2}\;and\;\mu_{0}=\sum_{j=0}^{t-1}\frac{jP_{j}}{\omega_{0}\left(t\right)},\;\mu_{1}=\sum_{j=t}^{L-1}\frac{jP_{j}}{\omega_{1}\left(t\right)}.$$

Global intensity means

(2) $$\mu_{T}=\omega_{0}\mu_{0}+\omega_{1}\mu_{1}$$

And where $\omega_{0}+\omega_{1}=1$, $\omega_{0}=\overset{t-1}{\underset{j=0}{\sum }}P_{j}$, $\omega_{1}=\overset{L-1}{\underset{j=t}{\sum }}P_{j}$.

The above method can be extended to multilevel thresholding problem, which is defined as

(3) $$\mathrm{Max}\left[f\left(t\right)=\varphi_{0}+\varphi_{1}+\ldots +\varphi_{n}\right]$$

where $\varphi_{n}=\omega_{n}{\left(\mu_{n}-\mu_{T}\right)}^{2}$ (*n* = 1, 2, 3, …).

While the number of thresholds n increases, an exhaustive search for n + 1 segment will result in exponential growth in computational time. To overcome this drawback, PSO is adopted as a solution for multilevel thresholding problems.

### 2.2. Intelligent Swarm Optimization Based on Particle Swarm

A PSO algorithm based on the simulation of bird individual hunting behavior can be used for intelligent swarm optimizations. It simulates the behavior of birds where each bird will make its contribution in the searching process depending upon its fitness. The overall fitness of the individuals is used for the identification of the food center which is considered as the best global estimation.

The implementation of the particle swarm optimization is detailed as follows:

Step 1 Initialize parameters $I_{max}$ (maximum number of iteration), M (number of particles), V (initial particle velocity) and X (initial particle position);
Step 2 Repeat the Step 3 to Step 7 for i = 1 to $I_{max}$;
Step 3 Calculate fitness for each particle;
Step 4 Update the best position of the particle $P_{id}^{t}$;
Step 5 Update the best position of the whole swarm $P_{gd}^{t}$;
Step 6 Update position X and velocity V;
Step 7 Determines whether meeting the termination conditions.

In PSO, an individual is used to represent the potential solution of the optimization problem. The position and velocity on each dimension are updated using the following two equations:

(4) $$v_{id}^{t+1}=wv_{id}^{t}+c_{1}rand_{1}\left(P_{id}^{t}-x_{id}^{t}\right)+c_{2}rand_{2}\left(P_{gd}^{t}-x_{id}^{t}\right)$$

(5) $$x_{id}^{t+1}=x_{id}^{t}+v_{id}^{t+1}$$

where d denotes one dimension of a particle, w is named as inertia weight, and $c_{1}$, $c_{2}$ are the acceleration coefficients uniformly distributed within (0,1). Generally, for each d ϵ 1, 2…, D, the value of $v_{id}^{t}$ is restricted within the range of $\left(-V_{max},V_{max}\right)$, and $x_{id}^{t}$ is between $(X_{min}$, $X_{max})$. Communal effects among the particles lead to movement since they always attempt to go to the best position. The velocity update equation can be divided into three parts. The first part is inertia or momentum, which reflects the motor habit of the particles. The second part is cognition, which reflects the memory of particles and denotes the trend to move close to the best historical position of the particle. The last part is social part, which reflects the collaboration between the particles and sharing of knowledge. It denotes the trend of the whole swarm to move close to the best position.

## 3. Proposed Method

**Figure 2:**
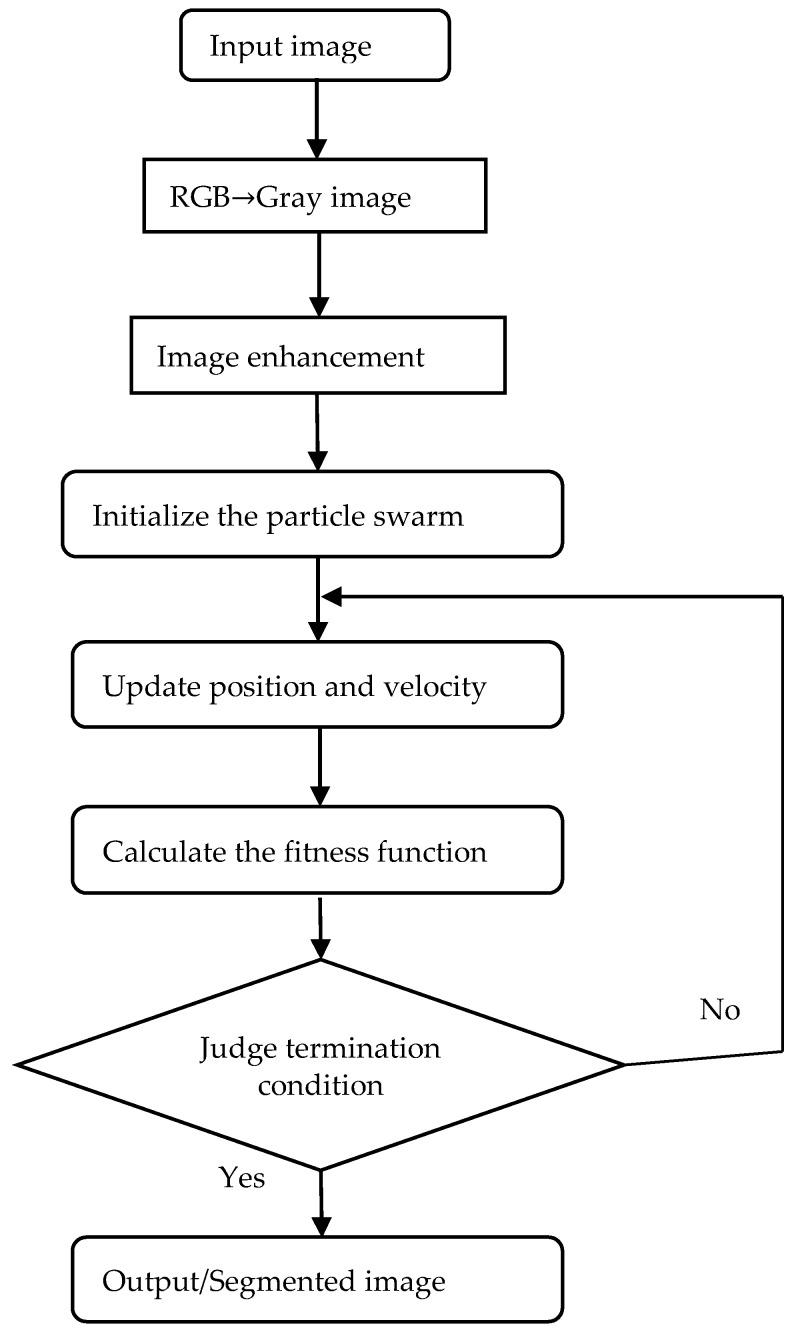
Graphical illustration of the proposed method.

A graphical illustration of the proposed method is given in Figure 2. In the proposed method, DPSO is utilized to find the optimal thresholds for segmentation of the image by maximizing the objective function of the improved Otsu algorithm. The step by step procedure for the proposed method is as follows:

Step I: Parameter initialization

(a) Initializes the maximum number of iterations $I_{max}$;
(b) Initializes velocity V and position X of particles;
(c) Set the lower boundary and upper boundary of V and X;
(d) Initializes the width of threshold band.

Step II: Image enhancement

(a) Histogram calculation;
(b) Histogram equalization.

The basic idea of histogram equalization is to put the histogram of the original image into a form of uniform distribution, thus increasing the pixel grayscale to a dynamic range to obtain the overall image contrast. The occurrence probability of the k-level grayscale of the original image can be denoted as

(6) $$p_{s}\left(g_{k}\right)=\frac{n_{k}}{n}\;0\leq g_{k}\leq 1,\;k=0,1,\ldots ,L-1$$

where $n_{k}$ is the independent variable and $p_{s}\left(g_{k}\right)$ is defined as the function is the histogram of the image.

The distribution of $s_{k}$ can be transformed into the uniform distribution of $t_{k}$ by using the cumulative distribution function:

(7) $$t_{k}=EH\left(s_{k}\right)=\overset{k}{\underset{i=0}{\sum }}\frac{n_{i}}{n}=\overset{k}{\underset{i=0}{\sum }}p_{s}\left(s_{i}\right)\;;\;0\leq s_{k}\leq 1,k=0,1,\ldots ,L-1$$

Step III: Fitness function calculation

Calculate the fitness of current particles’ position using the improved Otsu objective function.

Due to the low contrast of the image and inconspicuous boundary line, a single threshold cannot separate the crystal image well. Therefore, a continuous threshold band is proposed for segmentation. Because the crystal image has the characteristic of continuous grey values, taking the threshold band as the boundary line would classify a small part of the crystal as the boundary line and make the overall division clearer.

Equation (1) can be rewritten as:

(8) $$\mathrm{Max}\left[f\left(band\right)=\varphi_{0}+\varphi_{1}\right]$$

Thus,

(9a) $$\omega_{0}=\sum_{j=0}^{band_{left}}P_{j},\;\omega_{1}=\sum_{j=band_{right}}^{L-1}P_{j}$$

(9b) $$\mu_{0}=\sum_{j=0}^{band_{left}}\frac{jP_{j}}{\omega_{0}\left(band\right)},\;\mu_{1}=\sum_{j=band_{right}}^{L-1}\frac{jP_{j}}{\omega_{1}\left(band\right)}$$

**Figure 3:**
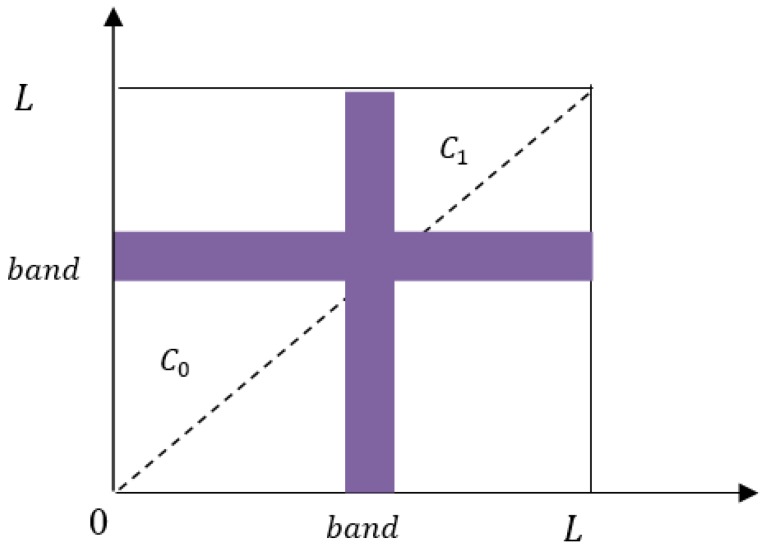
Improved Otsu method diagram.

The band is in the range of [$band_{left}$, $band_{right}$] and the image is divided into two categories, $C_{0}$ and $C_{1}$, according to the threshold band. $C_{0}$ and $C_{1}$ are in the range of [0, $band_{left}]$ and [$band_{right}$, L−1], respectively. As shown in Figure 3, $C_{0}$ and $C_{1}$ correspond to the background and target, and other areas represent noise and edges.

Step IV: Update position and velocity for each particle (using Equations (4) and (5))

The inertial coefficient is a key parameter for PSO performance. The fixed inertial parameter will cause the algorithm to converge to the local optimal solution. Moreover, the linear reduction of the inertial parameter will cause the algorithm to miss the global optimal solution. Hence, in the proposed method, the ω in Equation (4) is adopted [30]:

(10) $$\omega =\{\begin{array}{ll} \omega_{max}-\frac{\left(\omega_{max}-\omega_{min}\right)\times \left(f_{i}-f_{min}\right)}{f_{avg}-f_{min}}, & f_{i}\leq f_{avg}\\ \omega_{min}, & f_{i}>f_{avg} \end{array}$$

where $\omega_{\max}$ and $\omega_{\min}$ denote maximum or minimum inertia coefficient, respectively; $f_{i}$ is the current adaptive value with particle i; and $f_{\mathrm{avg}}$ is the current average fitness for all particles; $f_{\min}$ is the minimum fitness for all particles. When the fitness values of all particles converge or tend to converge, the inertia weight ω is automatically increased. On the other hand, when the fitness value of all particles scattered, the inertia weight value is automatically reduced. ω here is called dynamic inertia weight because the inertia weight is dynamically changed along with the fitness value. The advantages of dynamic inertia weight are shown in [30].

Also, in [30], dynamic learning factors are proposed. The selection of learning factors will also directly affect the output results. Therefore, it is necessary to select the appropriate learning factors in the optimizing process. The calculation formula is

(11) $$c_{1}=c_{1,beg}+\frac{c_{1,end}-c_{1,beg}}{I_{max}}\times I$$

(12) $$c_{2}=c_{2,beg}+\frac{c_{2,end}-c_{2,beg}}{I_{max}}\times I$$

where $c_{1,\;beg}>c_{2,\;beg}$; $c_{2,end}<c_{1,end}$, $I_{max}$ denotes the maximum number of iterations. $c_{1}$ and $c_{2}$ are automatically become larger or smaller along with time. Thus, at the initial stage of optimization, particles have strong self-learning ability and weak collective learning ability, enhancing the algorithm’s global search capability. In the later stages of optimization, the particles will have weak self-learning capability and strong social-learning ability so that the convergence to the global optimal solution can be accelerated.

Step V: Calculate fitness for each particle after updating.
Step VI: If the number of iterations reaches the maximum go to Step VII. Otherwise go to Step IV.
Step VII: Select the threshold band associated with the overall best particles.

Besides the Otsu objective function, regional consistency is also adopted to judge the effect of image segmentation. The interior of the region formed by image segmentation is similar, and the quality of the segmentation can be described by the degree of uniformity within each region. The ith region is represented by $R_{i}$, $A_{i}$ stands for its area, and f(x,y) represents the pixel value of the segmentation image with coordinate as (x,y). Then, the internal uniformity of the segmented image can be expressed as:

(13) $$UM=1-\frac{1}{C}\sum \left\{\underset{\left(x,y\in R_{i}\right)}{\sum }{\left[f\left(x,y\right)-\frac{1}{A_{i}}\underset{\left(x,y\in R_{i}\right)}{\sum }f\left(x,y\right)\right]}^{2}\right\}$$

where C is the total number of pixels in the image, UM ranges in (0,1). The larger the UM is, the better the uniformity within the region is.

## 4. Experiments and Results

### 4.1. Experimental Setup

In order to verify the performance of the proposed algorithm, a crystal growth image is taken as the object, and the DPSO-based image thresholding algorithm is validated through simulations in MATLAB on a computer with the following configuration:

Intel^(R)^ Core^(TM)^ i7-6700 CPU @ 3.40 GHz and 8 GB of memory with Microsoft Windows 10 operating system, produced by Legend Corp., Beijing, China.

**Figure 4:**
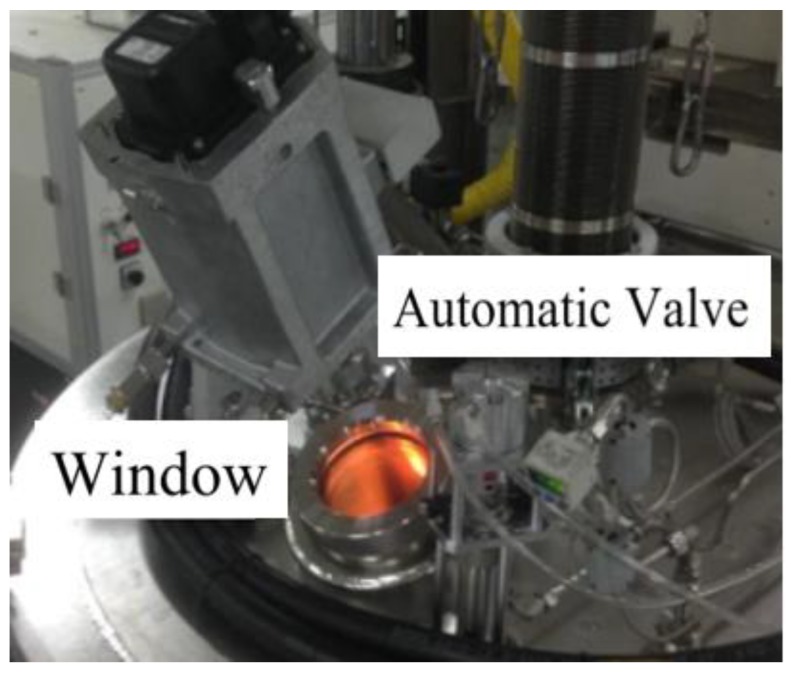
Crystal images acquisition device.

**Figure 5:**
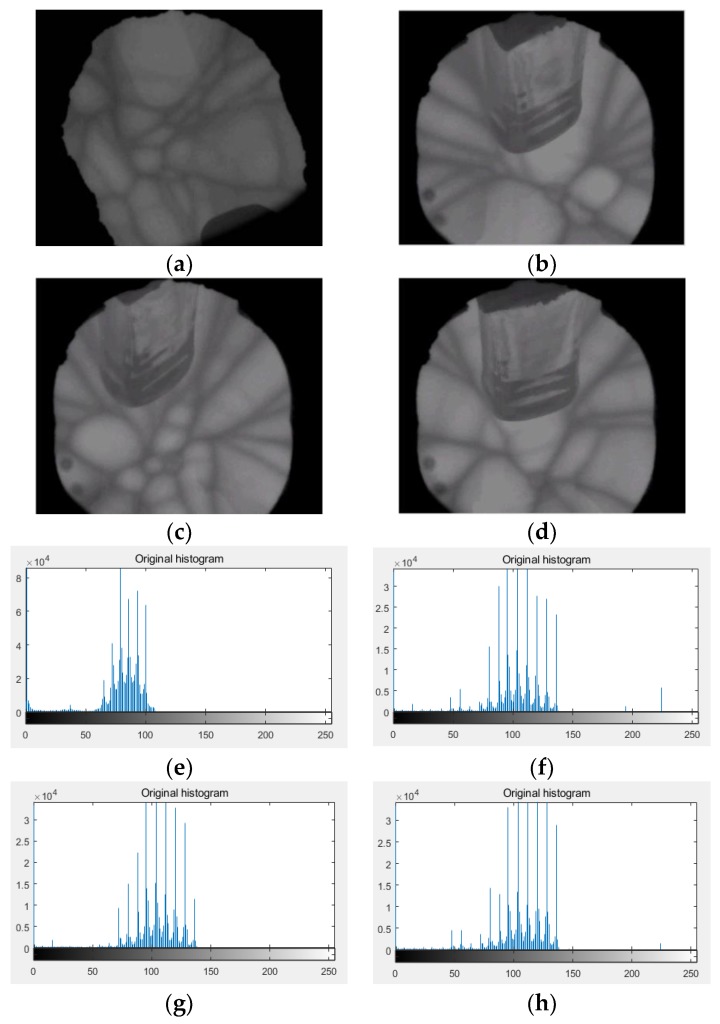
(**a**–**d**) are the original images of crystal growth images, (**e**–**h**) are the histograms of the original images.

The objective values along with the corresponding thresholds and CPU time obtained using the proposed method were compared to already reported multilevel thresholding algorithms, such as the BF, PSO, and GA algorithms. The input images are obtained by CCD as shown in Figure 4, namely Crystal1, Crystal2, Crystal3, and Crystal4, and their histograms are shown in Figure 5. All the images are of size 512 × 512.

### 4.2. Parameter Setting

The parameters used in the proposed DPSO algorithm are shown in Table 1. The parameters used for other methods (PSO, GA, BF) are shown in Table 2, Table 3 and Table 4 [28]. To evaluate the performance of the proposed algorithm, Otsu’s objective functions are considered.

### 4.3. Results and Discussion

In this study, to validate the efficiency of the proposed image segmentation algorithm for crystal growth, a set of experiments were conducted on a large set of real crystal growth images from different growth stages obtained from CCD. As shown in Figure 5, the dataset included four original crystal growth images. The grayscale distribution of the crystal image is most between [50,150] from the histogram, which results from the low contrast of crystal images.

**Figure 6:**
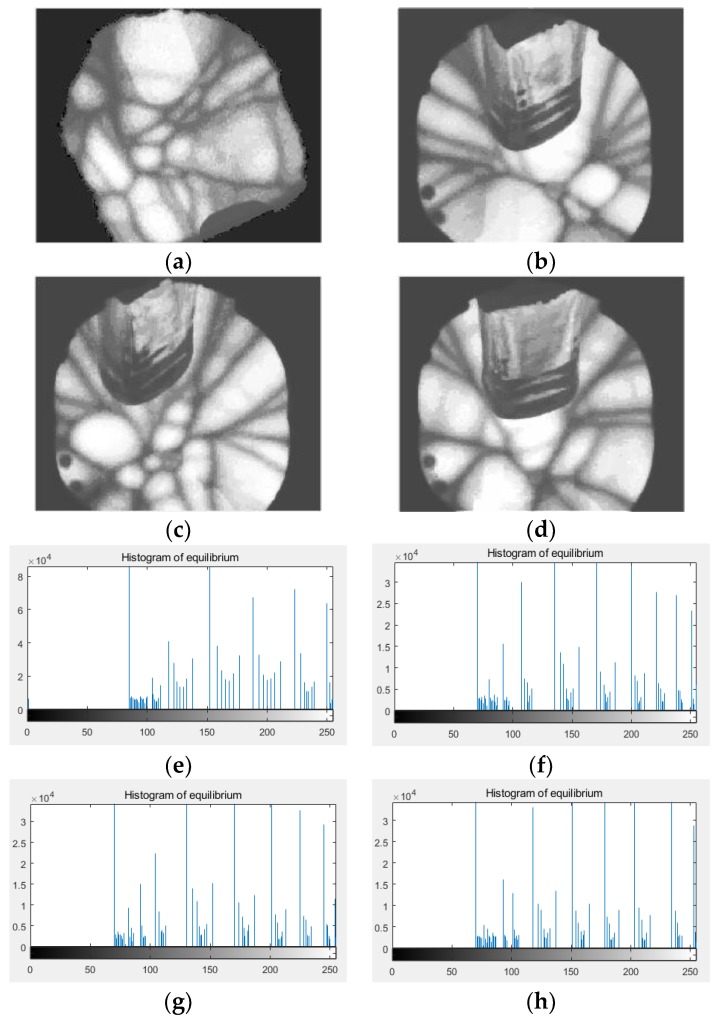
(**a**–**d**) are images after image enhancement, (**e**–**h**) are the histograms of images after image enhancement.

In order to enlarge the grayscale difference for segmenting, histogram equalization is used for image enhancement. The images after image enhancement and their histograms are given in Figure 6. Obviously, crystals and boundaries are more pronounced after image enhancement. What’s more, the histogram shows that the gray value of the whole image is divided into several parts, and the number of gray values in each part is about 20–30. In the proposed method, 25 continuous gray values are taken as the threshold bands, as too many gray values can lead to excessive defects in crystal images.

In Table 5, the comparisons of the proposed method with previous segmentation methods (PSO, BF, and GA) are shown. All experiments were conducted by the system with the configuration set up described in Section 4.2.

The objective function values obtained by the Otsu-based DPSO, PSO, BF, and GA algorithms are presented in Table 5. The threshold values and threshold band obtained by the Otsu-based DPSO, PSO, BF, and GA algorithms are presented in Table 6. A higher value of objective function leads to a better visual effect for the segmented image. The experimental results indicate that the proposed method yields the highest objective values compared to BF, PSO, and GA.

Table 7 illustrates the computational efficiency of the proposed method. The DPSO-based multilevel thresholding algorithm is compared with the existing bio-inspired methods in terms of convergence time (CPU time taken in seconds on an average). From Table 7, it is evident that the proposed DPSO-based multilevel thresholding method is much faster than the other bio-inspired algorithms. Table 8 shows the internal uniformity obtained by the DPSO-based method with other bio-inspired multilevel methods. According to Equation (13), values obtained by DPSO-based method is larger than PSO, BF, GA algorithms. Thus, DPSO-based method has better regional consistency.

**Figure 7:**
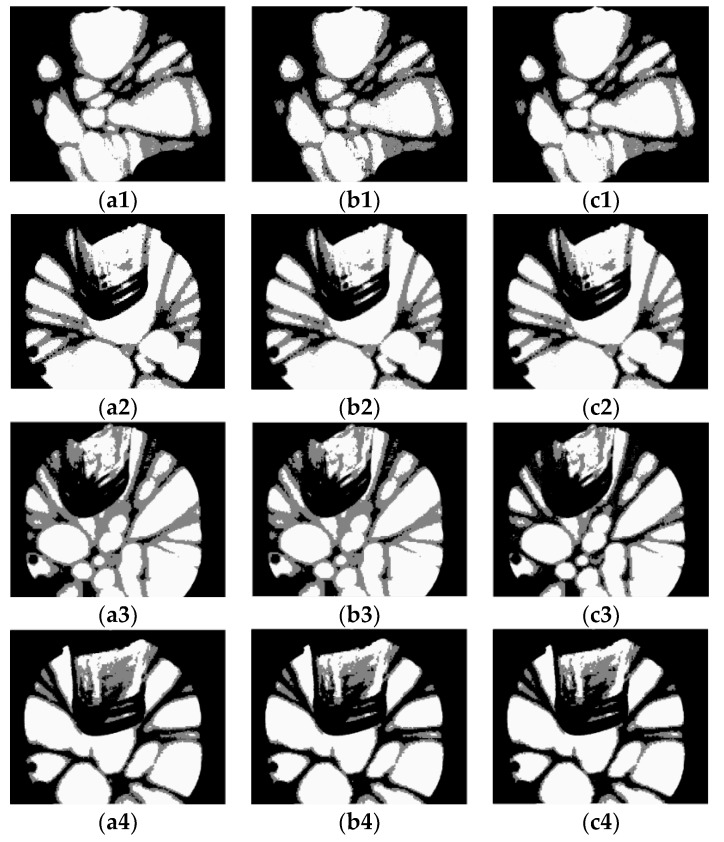
Segmented images obtained by the Otsu-based PSO, BF, and GA methods ((**a1**–**a4**, **b1**–**b4**, **c1**–**c4**) represent PSO, BF, and GA, respectively).

**Figure 8:**
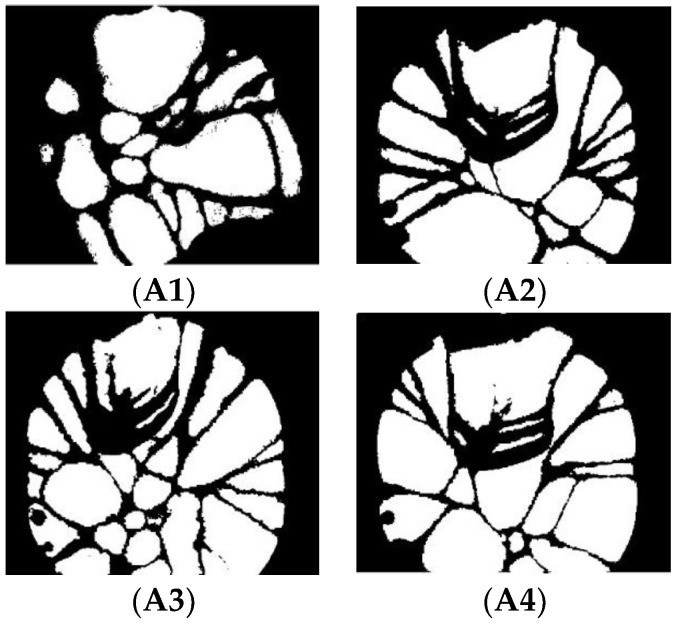
(**A1**–**A4**) are segmented images of a–d obtained by Otsu-based DPSO method.

The segmented images (Crystal1, Crystal2, Crystal3, and Crystal4) of the Otsu-based and bio-inspired methods are shown in Figure 7. The images of the Otsu-based DPSO method are shown in Figure 8. It is evident that the DPSO-based segmentation has the highest performance among all the investigated methods. Although the segmented images of the Otsu-based bio-inspired methods show most of the crystals, a small portion of the boundary is incorrectly classified as crystal due to signal threshold value. However, the threshold band is used as the boundary in the proposed method. The result shows that using the threshold band would classify a small part of the crystal as the boundary line but make the overall division clearer.

## 5. Conclusions

An improved image segmentation algorithm based on dynamic particle swarm optimization (DPSO) is proposed in this paper. Images of the melting interface are always obscure and low contrast due to the uneven and random interference from the heater. This means the separation lines of textures do not have the same gray degree. Hence, intelligent swarm optimization was introduced to solve the segmentation problem. In addition, using a threshold band as the boundary was adopted rather than multilevel thresholding in traditional methods for segmentation. Experimental results show that the proposed method based on DPSO gives better performance compared with several typical methods.

## Figures and Tables

**Table 1:** Parameters for DPSO.

Parameters	Value
Size of the Population (N)	25
Iteration Number	100
Inertia Weight ($\omega_{\max}$)	0.9
Inertia Weight ($\omega_{\min}$)	0.4
PSO random parameter	$c_{1,\mathrm{beg}}=c_{2,\mathrm{end}}=$ 3
	$c_{2,\mathrm{beg}}=c_{1,\mathrm{end}}=$ 0.5
Width of threshold band	25

**Table 2:** Parameters for PSO.

Parameters	Value
Size of the Population(N)	25
Iteration Number	100
Inertia Weight ($\omega_{\max}$)	0.9
Inertia Weight ($\omega_{\min}$)	0.4
PSO random parameter	C1 = 2
	C2 = 2

**Table 3:** Parameters for GA.

Parameters	Value
Size of the Population (N)	20
Iteration Number	100
Probability of Crossover (Pc)	0.8
Probability of Mutation (Pm)	0.05

**Table 4:** Parameters for BF.

Parameters	Value
Bacterium Number	40
Chemotactic Steps Number	10
Length of Swimming	10
Reproduction Steps Number	4
Elimination of Dispersal Events Number	2
Attractant Depth	0.1
Attract Width	0.2
Repellent Height	0.1
Repellent Width	10
Elimination and Dispersal Probability	0.02

**Table 5:** Comparative analysis of objective function values obtained by the Otsu-based DPSO method with other bio-inspired multilevel thresholding methods.

Test Images	Objective Values				
	DPSO	No. of Thresholds	PSO	BF	GA
Crystal1	1834.8	2	1821.4	1820.3	1806.9
Crystal2	1827.5	2	1814.2	1816.2	1808.0
Crystal3	1856.2	2	1829.6	1838.5	1820.3
Crystal4	1814.3	2	1804.3	1813.8	1795.0

**Table 6:** Comparative analysis of threshold values obtained by the Otsu-based DPSO method with other bio-inspired multilevel thresholding methods.

Test Images	Threshold Band	Threshold Values			
	DPSO	No. of Thresholds	PSO	BF	GA
Crystal1	146–171	2	130,164	131,163	130,164
Crystal2	142–167	2	135,161	135,162	135,161
Crystal3	145–170	2	132,160	132,160	133,160
Crystal4	143–168	2	135,162	135,163	134,163

**Table 7:** Comparative analysis of average CPU time obtained by the DPSO-based method with other bio-inspired multilevel thresholding methods.

Test Images	No. of Thresholds	CPU Time (s)			
		DPSO	PSO	BF	GA
Crystal1	2	3.1406	3.5769	3.2974	3.9643
Crystal2	2	2.9996	3.3268	3.1255	3.7584
Crystal3	2	2.8749	3.2646	3.0967	3.6843
Crystal4	2	2.8438	3.1539	2.9836	3.5288

**Table 8:** Comparative analysis of internal uniformity obtained by the DPSO-based method with other bio-inspired multilevel methods.

Test Images	Internal Uniformity			
	DPSO	PSO	BF	GA
Crystal1	0.6704	0.5742	0.5967	0.5721
Crystal2	0.7533	0.6445	0.6576	0.6419
Crystal3	0.7417	0.6525	0.6619	0.6541
Crystal4	0.7608	0.6129	0.6237	0.6113
